# Axial Crushing Behavior of EPP Foam-Filled Nested GFRP Tubes

**DOI:** 10.3390/polym18212661

**Published:** 2026-10-27

**Authors:** Mehmet İskender Özsoy, Muhammet Muaz Yalçın

**Affiliations:** Department of Mechanical Engineering, Faculty of Engineering, Sakarya University, 54187 Sakarya, Türkiye; iozsoy@sakarya.edu.tr

**Keywords:** GFRP, EPP foam, nested tube, axial compression, energy absorption

## Abstract

Polymer matrix composites are used in many components in the automotive sector due to their high specific mechanical properties, and their use in energy-absorbing structures provides significant reductions in vehicle weight. In this study, the energy absorption behavior and load-carrying capacity of nested glass fiber reinforced epoxy composite (GFRP) tubes with and without expanded polypropylene (EPP) foam filling were investigated under quasi-static axial loading. Three nesting arrangements of three concentric tubes were tested: equal-length, short-to-long and long-to-short. Foam filling increased the load-carrying capacity and the absorbed energy of all nested configurations. For the equal-length configuration, EPP foam filling increased the mean crushing force and absorbed energy by approximately 34%, while the specific energy absorption (SEA) improved by about 12%. In the short-to-long configuration, foam filling increased the absorbed energy by approximately 30%, whereas in the long-to-short configuration the increase was approximately 19%; however, the SEA increased by only 6% in the short-to-long configuration and remained practically unchanged in the long-to-short configuration, and the maximum force increased by 24–40% with foam filling. Among the investigated arrangements, the equal-length nested tubes exhibited the highest energy absorption, while the short-to-long configuration provided the lowest initial force and a stepwise load progression due to sequential tube activation. The long-to-short configuration demonstrated intermediate behavior with relatively higher force levels and moderate energy absorption. The results indicate that both foam filling and nesting arrangement govern the quasi-static crushing response of nested composite tubes and that the gain in absorbed energy due to foam filling must be weighed against the added mass and the higher transmitted force.

## 1. Introduction

Using composite materials in structural components has great importance for achieving lightness, durability, and energy efficiency by significantly reducing vehicle weight, fuel consumption, and emissions while also providing high strength and stiffness [1,2,3,4]. Nowadays, researchers are conducting design and production studies to improve the crashworthiness behavior of composite tubes. The prominent parameters among these are the fiber type, fiber sequence and orientation, effect of the matrix, tube geometry and filler materials used in the tubes [5,6]. In this context, glass fibers especially stand out with low production cost and design flexibility, enabling a wide range of engineering applications while providing high impact strength, low thermal expansion coefficient, and corrosion and chemical resistance properties [7,8,9]. Guo et al. [10] investigated the crashworthiness behavior of single-fiber, double-fiber and triple-fiber epoxy composite corrugated structures made of glass, carbon and aramid fibers under axial load. Accordingly, they stated that fiber hybridization and fiber alignment effects are effective on impact resistance and failure modes. Glass fiber showed the highest specific energy absorption/cost due to its mechanical behavior and lower cost. Also, glass fiber/carbon fiber and glass fiber/aramid fiber hybrid composites exhibit balancing properties in terms of weight, performance and cost. Khan et al. [11] studied the fiber orientation angle effect on the axial crushing behavior of filament wound glass fiber epoxy/polyvinyl chloride hybrid composite tubes. Accordingly, the load bearing and energy dissipation behavior of the tubes improved with increasing fiber orientation. Hegazy et al. [12] studied the energy dissipation behavior of nano carbon-filled glass fiber reinforced epoxy composite tubes. Mat et al. [13] carried out the quasi-static axial compression loading of wrapping polyester matrix-composites of glass fiber, carbon fiber and their hybrids on aluminum tubes. The axial rigidity of aluminum tubes increased with fiber reinforcement, and since buckling was delayed, the crashworthiness of aluminum/composite tubes improved.

Additionally, the researchers investigated the influence of internal core structure geometries on the mechanical behavior of energy absorbers: Mert et al. [14] investigated the quasi-static axial crushing behavior of cylinders made of aluminum, glass fiber-reinforced plastic, and hybrid structures combining these two materials, as well as hybrid cylinders with an aluminum honeycomb core. In hybrid tubes, the presence of the aluminum body prevented the composite layer from folding inward, leading instead to longitudinal cracking and outward splaying; meanwhile, although the addition of an aluminum honeycomb core resulted in a marginal increase in crushing efficiency, the added weight reduced the energy absorbed per unit mass and failed to yield the expected improvement in crashworthiness. Hussain et al. [15] experimentally and statistically investigated the energy absorption capacities of annular sandwich composite tubes manufactured from short carbon fiber-reinforced polyamide using additive manufacturing. During the design process, the number of internal supports, the number of concentric rings, and the wall thickness were selected as variables, and the effects of these parameters on performance were optimized. Torkian et al. [16] manufactured a lightweight, impact-absorbing model consisting of three concentric, nested glass-fiber-reinforced composite tubes, with polyurethane foam filling the spaces between them. It was determined that nesting the tubes increased energy absorption capacity by 2.5 to 5 times compared to single-tube structures; furthermore, the foam filling boosted this value by an additional 28% by preventing premature buckling and delamination. Khan et al. [17] experimentally evaluated six geometric designs incorporating carbon-fiber outer layers, cellular polymer fillers, and internal metallic channels for mechanical resistance and impact-damping capabilities under quasi-static axial compressive loads. Zakki and Windyandari [18] investigated the internal cross-sectional geometries (circular, hexagonal/honeycomb, square, triangular) of GFRP panels through dynamic analysis and determined the effect of cellular shape selection on impact resistance. Chen et al. [19] investigated the axial crushing and impact energy absorption performance of multi-compartment carbon-reinforced composite tubes. The effects of internal partition fiber angles, cell counts, and internal dimensional ratios on the structure’s load-bearing stability were evaluated. The results revealed that a right-angle ply orientation yielded the highest mean crushing force and specific energy absorption, while the multi-cellular architecture significantly enhanced deformation resistance through mechanical interaction between the sub-compartments. Paz et al. [20] investigated the crashworthiness of square steel energy-absorbing elements featuring an internal glass-fiber-reinforced honeycomb structure. The study focused on optimizing mass, energy absorption, and peak reaction force, while treating steel and honeycomb wall thicknesses, cell size, and the height of the internal structure as design variables. It demonstrated that simultaneous crushing of the outer metal tube and the inner cellular structure resulted in a 22% increase in energy absorption due to a positive interaction effect, exceeding the sum of the individual components. Ghimire et al. [21] developed a nested cellular structure design in which smaller secondary hexagonal units are embedded within the main pores, and these designs were fabricated using 3D printers with flexible polymer materials. The analyses revealed that, compared to conventional honeycomb structures, this developed hierarchical design achieved a significant increase on doubling the compressive strength and enhancing energy absorption performance per unit mass by up to fivefold, while offering a two-stage impact protection mechanism on axial compression.

On the other hand, foams are widely used in the tubes to improve the crushing behavior. Foams’ cellular structure allows for controlled deformation, suppressing sudden buckling in the tube walls and promoting a more stable crushing process [22,23]. Despite their low density, their ability to absorb high deformation energy makes these materials highly advantageous in energy-absorbing systems where lightweight performance is critical [24,25,26]. Babbage and Mallick [27] investigated the axial crushing behavior of circular and square section aluminum composite hybrid tubes by wrapping different numbers and angles of glass fiber/epoxy composite layers on them. They also filled the tubes with epoxy foam and investigated the effects on the energy absorption capacity of the tubes. Wang et al. [28] investigated the effects of composite layer thickness, tube diameter, foam density and fiber orientation angles on energy absorption behavior in polyurethane foam filled GFRP tubes. Accordingly, higher compressive rigidity and higher energy absorption capacity were achieved with foam filled tubes. Similarly, increasing the ply number of the composites and increasing the tube diameter also increased the energy absorption capacity. Kathiresan and Manisekar [29] studied the axial crushing behavior of aluminum and aluminum/glass fiber epoxy hybrid composite conical frusta under impact. They stated that the buckling in aluminum tubes was limited by the fiber wrapping on the tube, and the load carrying and energy absorption capabilities increased. Jin et al. [30] investigated the load bearing and energy absorption of GFRP tubes under axial crushing by forming frame structures with GFRP rods from inside and filling them with synthetic foam. Buckling strength, axial rigidity and energy dissipation properties of the composites are increased by filling the tubes with foam structures from inside and with internal FRP supports.

Nested, bitubular and multi-tube configurations have also been investigated to increase the energy absorption of tubular absorbers. Aktay et al. [31] investigated experimentally and numerically the quasi-static crushing of empty and foam-filled single, bitubular and packed multi-tube aluminum configurations. Azarakhsh et al. [32] analyzed the axial crushing of empty and foam-filled brass bitubular tubes, and Djamaluddin et al. [33] optimized foam-filled double circular tubes under axial and oblique impact loading. For composite and hybrid tubes, Güden et al. [34] reported that aluminum foam filling stabilized the progressive crushing of glass fiber/polyester composite tubes, although the crushing load did not exceed the sum of the crushing loads of the empty tube and the foam. Khalid and Morsidi [35] studied the axial crushing of empty and polyurethane foam-filled jute–glass/epoxy bi-tubes, and Khan et al. [36] showed that adding intermediate tubes to hollow composite tubes significantly improved their load-bearing and energy absorption capabilities, whereas polyurethane foam filling increased the peak load, mean load and absorbed energy but did not consistently improve the SEA because of the added mass.

As seen in the literature, studies are carried out on energy absorbers under the effects of different fiber arrangements, tube geometries and test conditions. Moreover, epoxy resins offer a wide range of uses in engineering applications due to their high strength and excellent adhesion to fiber materials, enabling strong bonding of fiber-matrix interfaces [37,38]. In addition, their high thermal properties allow the composite components to perform reliably at elevated temperatures, making them well-suited for demanding automotive and aviation applications [39,40].

Despite the extensive literature on foam-filled single composite tubes [27,28,41] and on foam-filled bitubular and multi-tube absorbers [31,32,33,35,36], experimental data on nested composite tubes in which the tube lengths are graded to produce a staged force–displacement response remain scarce. In our previous studies, single EPP foam-filled CFRP tubes were investigated under axial compression [41], and single EPP foam-filled CFRP and GFRP tubes were investigated under lateral compression [42]; however, several tubes acting together, length grading and foam placed between nested tubes were not considered. In particular, it is not clear how the tubes of a length-graded nested GFRP absorber are engaged during crushing, how the nesting arrangement affects the force–displacement response and the energy absorbed within a given stroke, and how EPP foam placed in the core and in the gaps between the tubes changes this response. The following hypotheses were therefore formulated: (H1) in length-graded nested GFRP tubes, the tubes are engaged at displacements equal to their height differences, producing a stepwise force response; (H2) EPP foam in the core and in the inter-tube gaps increases the crushing force of the nested tubes by restraining the inward movement of the crushed wall material; and (H3) within a given stroke, the arrangement in which the largest-diameter tube is engaged first absorbs more energy, whereas the arrangement in which the smallest tube is engaged first gives the lowest initial force. To test these hypotheses, the energy absorption behavior of three-tube nested epoxy-matrix GFRP absorbers with equal-length, short-to-long and long-to-short arrangements was investigated experimentally with and without EPP foam filling under quasi-static axial loading.

## 2. Materials and Methods

Plain-woven E-glass fabric (*A_w_* = 200 g/m^2^) was used as the reinforcement (supplied by Dost Kimya Company, İstanbul, Türkiye), and Sika CR80 epoxy resin and Sika CH80-2 hardener were used as matrix materials (Tekno Endüstriyel Kimyasallar Company, İstanbul, Türkiye), respectively, mixed at a weight ratio of 100:80. EPP foam, with a density of 30 kg/m^3^ (0.03 g/cm^3^), supplied by Atermit Company, Sakarya, Türkiye, was used as the filling material inside the tubes. Cylindrical tubes were wrapped onto mandrels using the hand-lay-up technique with 7 (*n*) plies of fabric, resulting in a wall thickness of (*t*) 1.54 mm. At each wrapping stage, resin impregnation was achieved using brushes, after which rollers were applied to eliminate entrapped air voids.

Composite tubes were kept in the oven at 60 °C for 4 h after waiting at room temperature for 24 h. Fiber volume fraction (*V_f_*) and theoretical density (*ρ_c_*) of the composite structures were determined based on the rule of mixtures [43,44]. For a tube with seven layers (*n* = 7) and a fabric areal weight (*A_w_*) of 200 g/m^2^, the total fiber mass per unit area was calculated as *W_f_* = 0.140 g/cm^2^ as given in Equation (1).

*W_f_* = *n* × *A_w_* (1)

Considering the wall thickness of the composite tubes is 1.54 mm and the glass fiber density is 2.59 g/cm^3^ (supplier data sheet), the fiber volume fraction was determined to be 35.10% (*V_f_* = 0.3510) in Equation (2).

(2) $$V_{f}=\frac{W_{f}}{\rho_{f}\times t}$$

With a resin density of 1.10 g/cm^3^ (supplier data sheet), the matrix volume fraction was calculated as 64.90% (*V_m_* = 0.6490) in Equation (3):

*V_m_* = 1 − *V_f_* (3)

and the theoretical density of the composite is obtained as 1.623 g/cm^3^ according to Equation (4).

*ρ_c_* = *V_f_* × *ρ_f_* + *V_m_* × *ρ_m_* (4)

**Figure 1:**
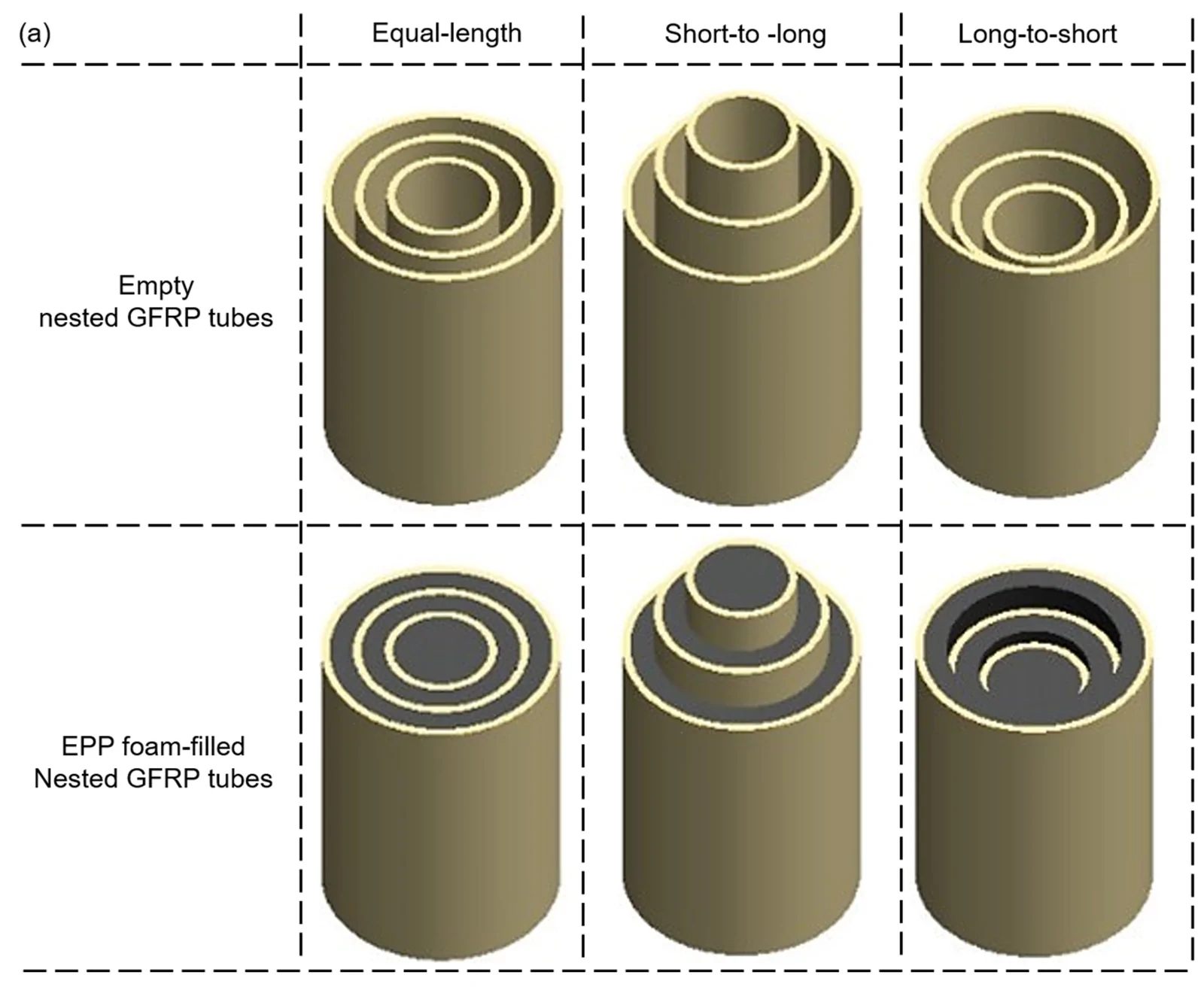

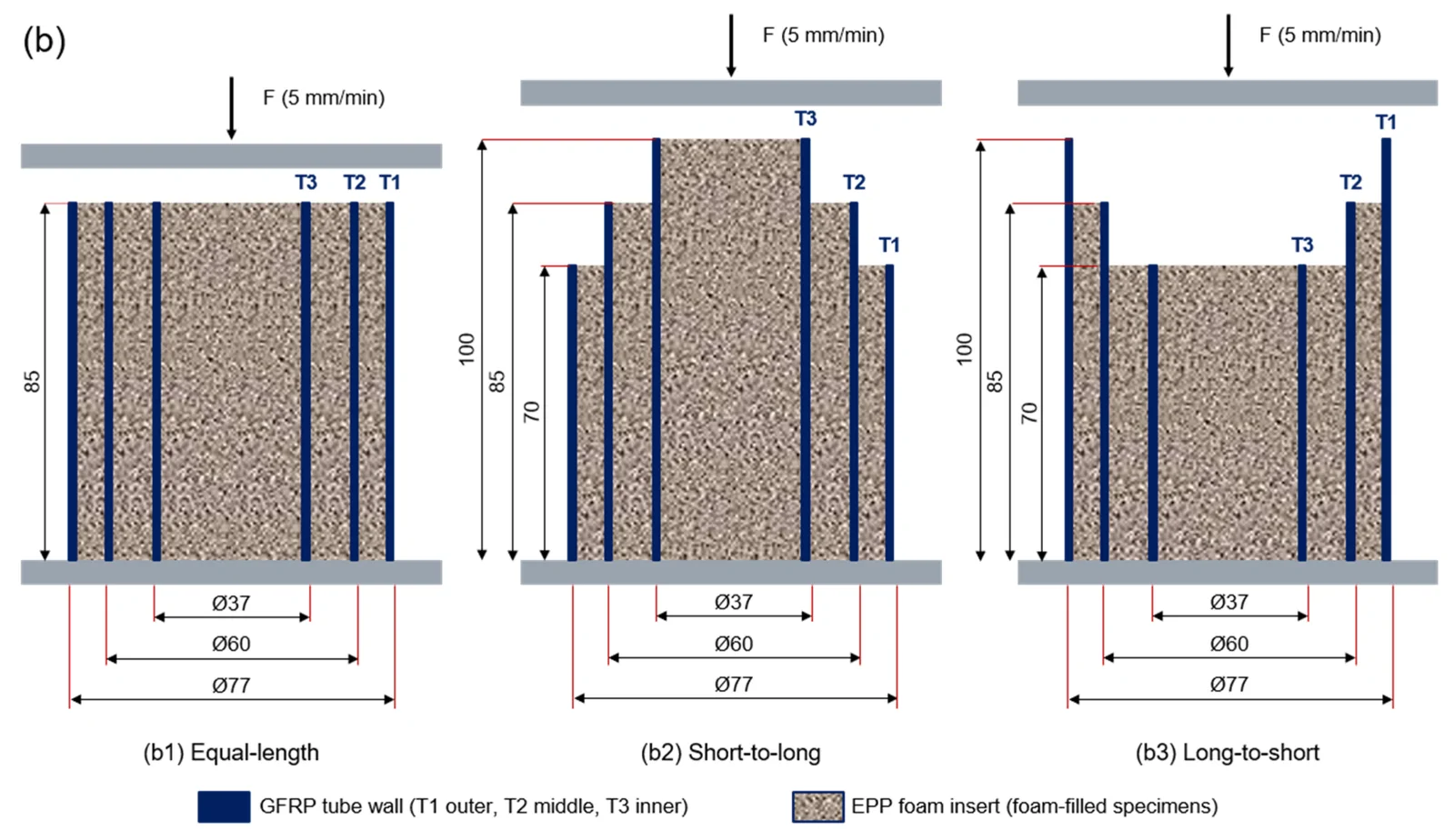
(**a**) Nested tube combinations of tubes (the empty nested tube samples are shown in the first row, and the foam-filled samples are shown in the second row; from left to right: equal-length, short-to-long, and long-to-short); (**b**) dimensioned schematic of the (**b1**) equal-length, (**b2**) short-to-long and (**b3**) long-to-short configurations (outer diameters and lengths in mm; T1: outer, T2: middle, T3: inner tube).

Tubes were prepared with outer diameters of 37, 60, and 77 mm and lengths of 70, 85, and 100 mm and were assembled concentrically into three nesting configurations (Figure 1 and Table 1): (i) equal-length, in which the three tubes have different diameters but the same length of 85 mm; (ii) short-to-long, in which the tube length increases from the outer to the inner tube (outer Ø77 × 70 mm, middle Ø60 × 85 mm and inner Ø37 × 100 mm), so that the inner tube protrudes furthest and is loaded first; and (iii) long-to-short, in which the tube length decreases from the outer to the inner tube (Ø77 × 100 mm, Ø60 × 85 mm and Ø37 × 70 mm), so that the outer tube is loaded first. To facilitate controlled initiation of axial crushing, the upper end of each tube was chamfered at 45° over a length of 1 mm. The tubes were not bonded to each other. In the foam-filled specimens, EPP inserts machined from the molded blocks filled the core of the inner tube and the annular gaps between the tubes; the inserts were machined to fit snugly and were pressed into place without any adhesive. Figure 1a shows the test groups of nested tube combinations, and Figure 1b shows a dimensioned schematic of the three configurations with the tube numbering, the foam locations and the loading direction.

### 2.1. Experimental Procedure

The quasi-static axial compression tests were carried out on an MTS universal testing machine with a maximum capacity of 100 kN at a crosshead speed of 5 mm/min, which is commonly used for quasi-static crushing tests [41,45,46]. No specific testing standard was followed; the procedure commonly used in the crashworthiness literature was adopted. The specimens were placed between flat, parallel steel platens, and a special centering fixture was used so that the nested tubes were in the same position in every test. The tests were terminated at a displacement of 45 mm, at which the force reached ≈96 kN in the foam-filled equal-length specimens, close to the capacity of the testing machine; continuing the test would have raised the force to levels that could damage the machine. For a consistent comparison, all crashworthiness indicators were evaluated over this 45 mm stroke for all specimens. Three specimens were tested for each configuration (18 tests in total).

### 2.2. Crashworthiness Parameters

**Figure 2:**
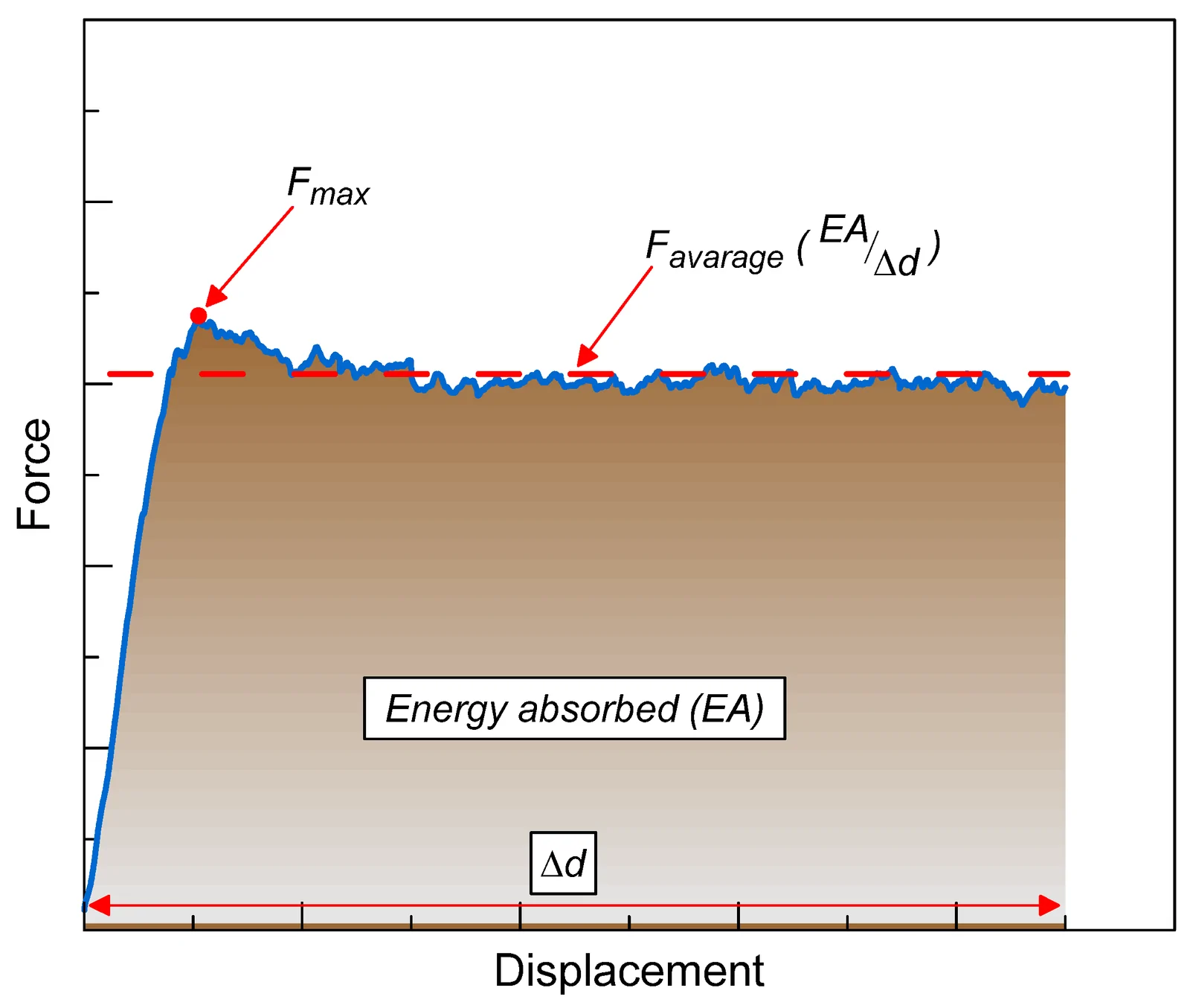
Force-displacement response of a tubular structure subjected to axial loading.

The force-displacement behavior of the thin-walled tubular structure under axial loading is shown in Figure 2. The force rises rapidly to a peak, then gradually decreases as the tube deforms. Tubes that exhibit higher peak forces can transfer impact loads without failing, which can lead to damage initiating directly in the main structure [41,47]. The first peak force is close to the mean force, thereby improving overall crashworthiness and indicating an optimal crash box [41,48]. The absorbed energy (AE) during axial crushing is determined from the area enclosed by the force-displacement curve shown in Figure 2.

The absorbed energy value is obtained using Equation (5), where F(x) is the instantaneous crushing force, x is the axial displacement and l is the crushing stroke (l = 45 mm in this study).

(5) $$\mathrm{AE}=\int_{0}^{l}F\left(x\right)\mathrm{dx}$$

The crashworthiness performance of energy-absorbing systems is typically characterized by parameters such as AE, SEA, and CFE [42,49]. These indicators are not defined by a specific standard but are widely used in the crashworthiness literature [48,49]. AE is the absorbed energy, and SEA denotes the energy absorbed per unit mass, as given in Equation (6), where m is the total mass of the specimen.

(6) $$\mathrm{SEA}=\frac{\mathrm{AE}}{m}$$

Crush force efficiency (CFE) represents the relationship between the mean crushing force and the global maximum force (F_max_) recorded over the crushing stroke, which is given in Equation (7) [42,50]. The mean crushing force (F_mean_) is computed as specified in Equation (8). CFE is a dimensionless quantity; since F_max_ is the global maximum force, the same definition applies to all configurations, including those in which the maximum force occurs late in the stroke.

(7) $$\mathrm{CFE}=\frac{F_{\mathrm{mean}}}{F_{\max}}$$

(8) $$F_{\mathrm{mean}}=\frac{\mathrm{AE}}{l}$$

## 3. Results and Discussion

### Axial Crushing Response of Nested GFRP Tubes

**Figure 3:**
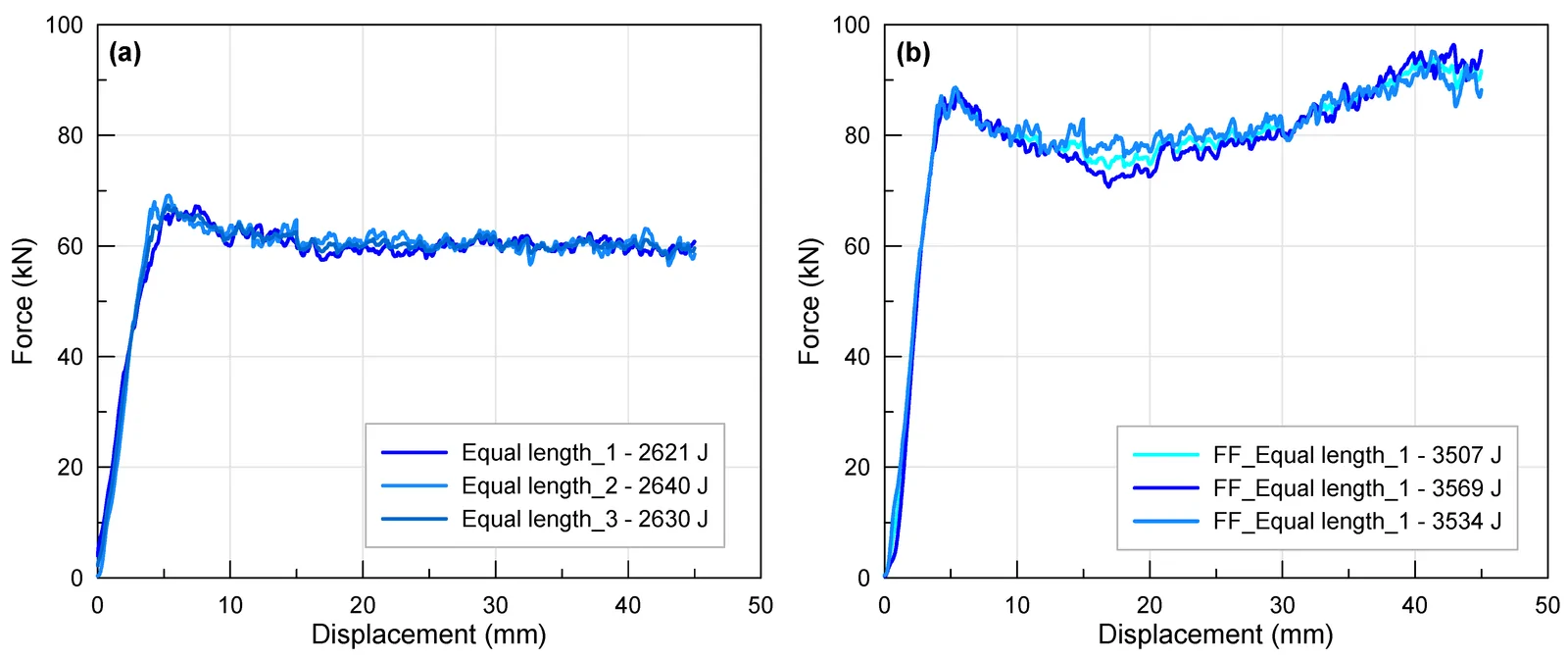
The force-displacement curves of the three repeated tests of (**a**) empty and (**b**) EPP foam-filled equal-length nested GFRP tubular structures (all GFRP tubes are 85 mm long).

The force–displacement curves presented in Figure 3a correspond to the axial compression tests conducted on the empty equal-length nested GFRP tubes. The results obtained from three repeated experiments are shown in the figure, and the strong overlap between the curves clearly indicates highly repeatable deformation behavior of the structures. At the initial stage of the test, the force-displacement curves exhibit a very steep increase, which corresponds to the elastic response of the tubes, during which no significant damage or instability is observed. At a displacement of approximately 5–6 mm, the force reaches its maximum value, with peak forces in the range of 67–69 kN. This peak force represents the critical load at which the initial damage and the onset of stable crushing mechanisms occur within the tubular structures. Following the maximum force, a slight reduction in force is observed, after which the curves enter a relatively stable plateau region extending approximately from 20 mm to 45 mm of displacement. Within this region, the force remains nearly constant, fluctuating between 58 and 62 kN. This behavior is characteristic of progressive crushing in composite tubes, in which energy is absorbed in a controlled and continuous manner. The minor oscillations observed along the plateau can be attributed to localized damage mechanisms such as fiber fracture, matrix cracking, and delamination. No pronounced increase in force is observed in the final stage of the test, which indicates that the empty specimens did not reach densification within the 45 mm stroke. The absorbed energy values for the three specimens are 2621 J, 2640 J, and 2630 J, respectively, yielding an average absorbed energy of 2630 J and an average mean crushing force of 58.5 kN. Figure 3b shows the three repeated tests of the foam-filled equal-length specimens, which also overlap closely (3507 J, 3569 J, and 3534 J; average 3537 J). After an initial peak of approximately 88 kN, the force remains at approximately 72–85 kN up to a displacement of about 30 mm and then rises to approximately 96 kN towards the end of the stroke. This rise is associated with the compaction of the foam and of the crushed debris between the tubes, i.e., the onset of densification, and it was the reason for terminating the tests at 45 mm (Section 2.1). In summary, the force-displacement curves indicate that both the empty and the foam-filled equal-length GFRP tubes exhibit excellent repeatability and stable progressive crushing behavior under axial compressive loading. The observed trends are consistent with the typical force-displacement responses reported in the literature for composite tubular structures.

**Figure 4:**
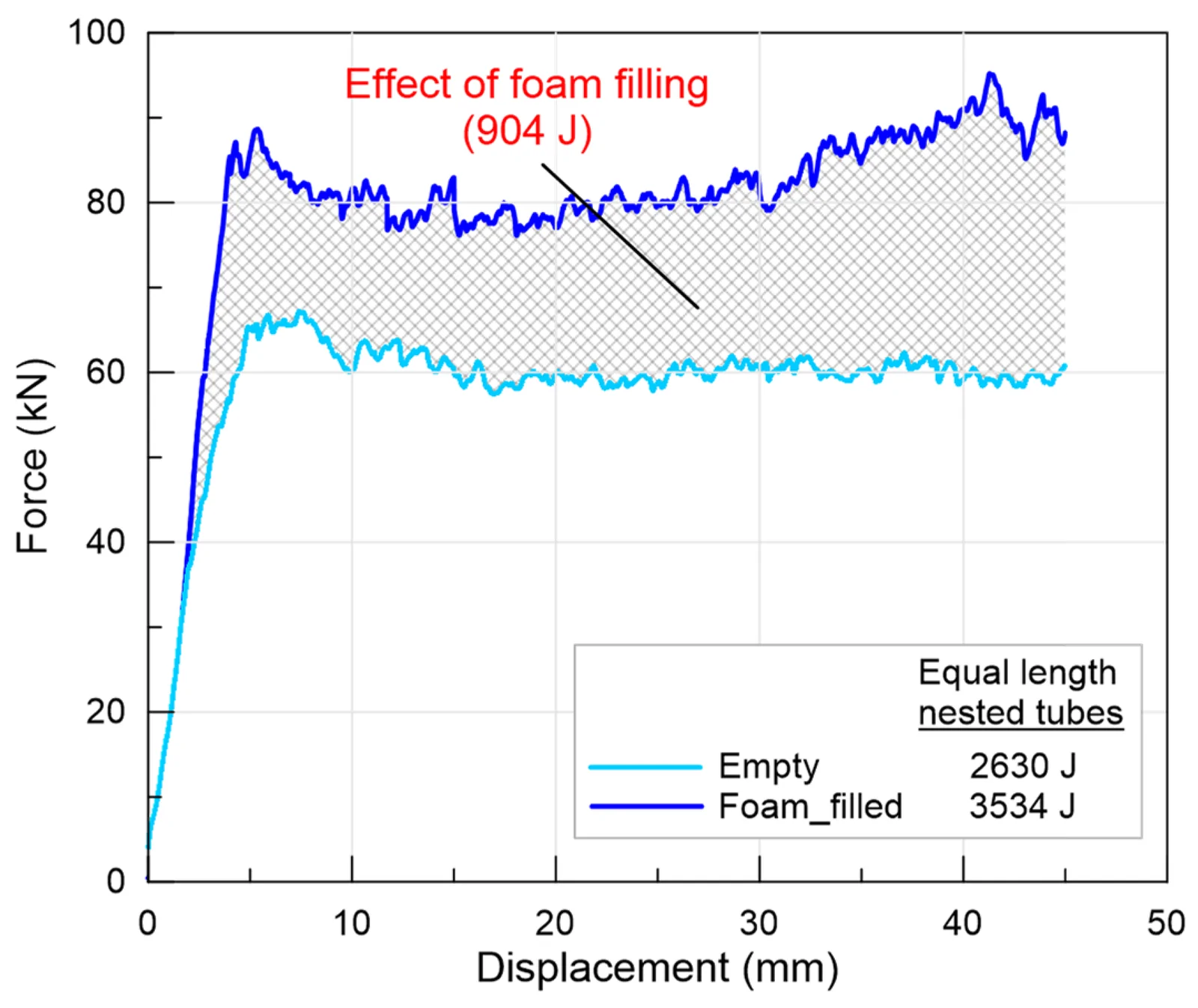
Representative force-displacement curves of empty and EPP foam-filled equal-length nested GFRP tubular structures (specimen 3 of each group); the shaded area shows the foam filling effect in terms of absorbed energy (all GFRP tubes are 85 mm long).

Figure 4 compares representative force-displacement responses of empty and expanded polypropylene (EPP) foam-filled equal-length nested GFRP tubes under quasi-static compression. Both configurations exhibit a similar initial elastic response; however, the foam-filled specimens demonstrate a steeper slope at the beginning of the curve, indicating a higher global structural stiffness. This behavior can be attributed to the internal support provided by the EPP foam, which restrains the inward movement of the GFRP tube walls during the early stages of deformation. Following the elastic regime, the empty GFRP tubes reach a relatively stable plateau load of approximately 60 kN. In contrast, the EPP foam-filled tubes sustain a higher plateau load in the range of 80–90 kN. The increase in mean crushing force suggests that the foam effectively restrains inward deformation of the tube walls. The slightly increased oscillations observed in the foam-filled response are likely associated with the interaction between the fracture mechanisms of GFRP (matrix cracking, fiber fracture, and delamination) and the progressive compaction of the EPP foam. From an energy absorption perspective, the representative foam-filled specimen absorbs 3534 J, whereas the empty specimen absorbs 2630 J, and the shaded area between the curves corresponds to 904 J. Based on the group averages, foam filling increased the absorbed energy from 2630 J to 3537 J, i.e., by approximately 34.5%. This increase reflects the foam filling effect, i.e., the energy absorbed by the foam together with its effect on the crushing of the GFRP walls, whose inward movement is restrained by the foam. Since the ring-shaped foam inserts buckled when tested alone, the contribution of the foam itself could not be separated from this foam–tube interaction. Nevertheless, the increased crushing force associated with foam filling should be carefully evaluated in applications where transmitted forces must be minimized; in the equal-length configuration, the maximum force increased from 68.2 kN to 95.5 kN. Overall, the results demonstrate that EPP foam-filled nested GFRP tubes exhibit a higher load-carrying capacity and absorb more energy than their empty counterparts.

**Figure 5:**
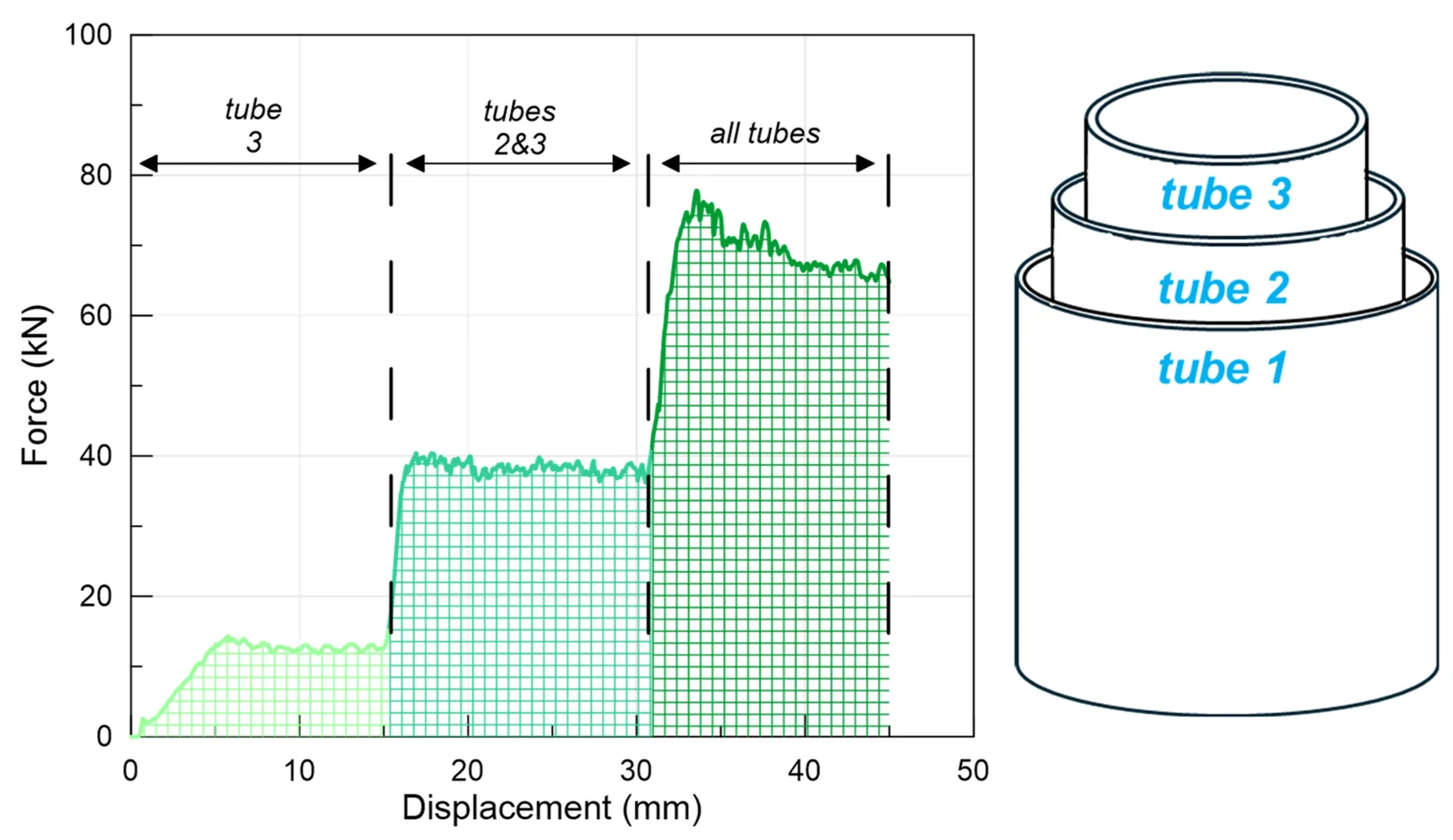
The stepwise force-displacement curve of the empty short-to-long nested GFRP tubular structure and the tube numbering (GFRP tubes were arranged concentrically, with the shorter tube positioned on the outer side and the longer tube placed on the inner side; tube 1: Ø77 × 70 mm, tube 2: Ø60 × 85 mm, tube 3: Ø37 × 100 mm).

The force–displacement curves presented in Figure 5 illustrate the axial compression response of GFRP tube assemblies arranged in a short-to-long length configuration and are directly comparable to the results previously obtained for the equal-length tube specimens. In contrast to the equal-length tubes, which exhibited a single dominant peak force followed by a relatively continuous plateau region, the short-to-long configuration displays a distinct stepwise force–displacement response. At the initial stage of loading, the force increases gradually and stabilizes at a relatively low level of approximately 12–14 kN within the first 8–10 mm of displacement. This behavior corresponds to the deformation of the longest, innermost tube (tube 3 in Figure 5), which protrudes furthest and is therefore the first structural element to engage under axial compression. Compared to the equal-length configuration, this initial force level is significantly lower, indicating a more progressive and less abrupt load introduction. At a displacement of approximately 15–17 mm, a pronounced and abrupt increase in force is observed, with the force rising to around 38–40 kN. This force jump marks the onset of deformation in the intermediate-length tube (tube 2). Following this increase, the force remains relatively stable over a displacement range up to approximately 28–30 mm, forming a plateau region similar in nature to the plateau observed in the equal-length specimens, though at a lower force level. This suggests that, while both configurations exhibit progressive crushing behavior, the equal-length tubes sustain higher mean crushing forces, whereas the short-to-long configuration distributes deformation over multiple stages. A second sharp increase in force occurs at approximately 30–32 mm of displacement, corresponding to the engagement of the shortest. The force rapidly reaches a maximum value of approximately 72–75 kN, which is comparable to the peak forces observed in the equal-length tube tests. However, unlike the equal-length specimens, in which the response was characterized by a single extended plateau, the short-to-long configuration reaches this force level only after sequential tube activation. Beyond this point, the force stabilizes within the range of 65–70 kN, indicating continued progressive crushing rather than immediate densification.

**Figure 6:**
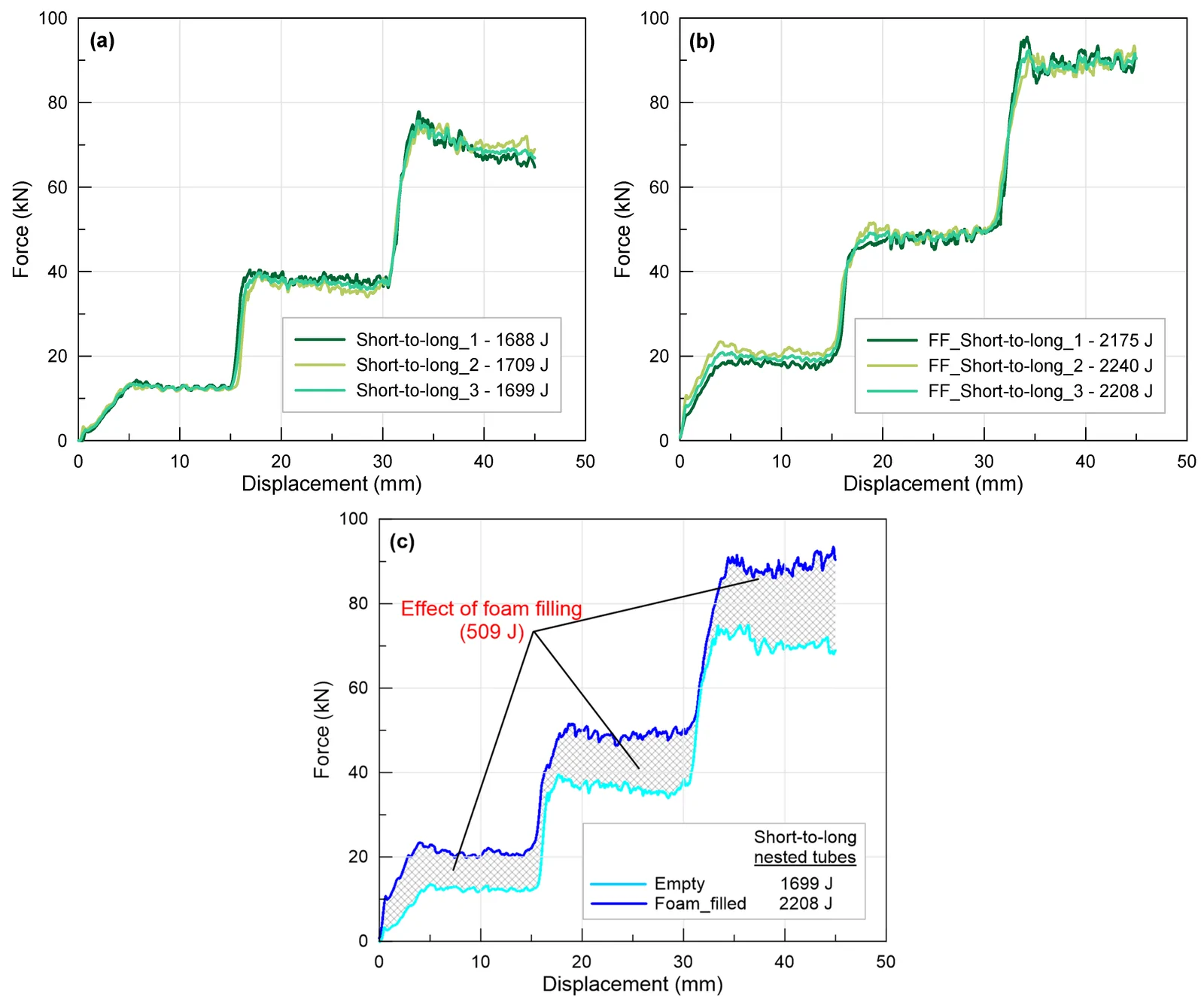
The force-displacement curves of the three repeated tests of (**a**) empty and (**b**) EPP foam-filled short-to-long nested GFRP tubular structures; (**c**) representative curves of empty and foam-filled nested samples (specimen 3 of each group) show the foam filling effect in terms of absorbed energy (GFRP tubes were arranged concentrically, with the shorter tube positioned on the outer side and the longer tube placed on the inner side; tube 1: Ø77 × 70 mm, tube 2: Ø60 × 85 mm, tube 3: Ø37 × 100 mm).

Figure 6 presents the axial force-displacement responses of the short-to-long nested GFRP tube configuration under quasi-static compression: the three repeated tests of the empty (Figure 6a) and foam-filled (Figure 6b) specimens and representative curves of both (Figure 6c). The repeated curves overlap closely, with absorbed energies of 1688, 1709, and 1699 J for the empty and 2175, 2240, and 2208 J for the foam-filled specimens. Consistent with the sequential activation mechanism described for Figure 5, the response exhibits a distinct stepwise crushing pattern, reflecting the progressive engagement of individual tubes during deformation. In both configurations, the initial stage is characterized by a relatively low and stable force plateau corresponding to the crushing of the innermost, longest tube. As displacement increases, the middle and outer tubes are engaged at approximately 15 and 30 mm, resulting in pronounced load increments and higher force plateaus. This behavior confirms that the nested architecture effectively distributes deformation over multiple stages, thereby promoting progressive energy absorption. Apart from the force steps caused by tube engagement, no sudden drops in force occur, indicating that the tubes are progressively crushed without global instability. A clear enhancement in load-carrying capacity is observed for the foam-filled configuration across all crushing stages. The foam-filled tubes consistently exhibit higher plateau forces compared to the empty system, which is attributed to the foam restraining the inward movement of the crushed wall material. Based on the group averages, the total absorbed energy increases from 1699 J for the empty system to 2208 J for the foam-filled system. This corresponds to an approximate 30% improvement in energy absorption capacity; the shaded area in Figure 6c (509 J) illustrates the foam-filling effect for the representative specimens. The elevated force levels in the foam-filled configuration correspond to a proportional increase in the mean crushing force (from 37.7 kN to 49.1 kN), directly contributing to the observed energy gain. However, the maximum force also increases (from 76.2 kN to 94.6 kN), and the CFE remains low (0.50–0.52) because of the stepwise force profile. Together with the analysis in Figure 5, these results show that foam filling increases the force at each crushing stage while preserving the controlled, stepwise load evolution of the nested design. The short-to-long configuration, therefore, provides a gradual, hierarchical force build-up with a low initial force, which may be advantageous in applications where such a build-up is desirable. Overall, when compared with the equal-length GFRP tube structures, the short-to-long configuration demonstrates a clear transition from a single-stage, high-force crushing response to a multi-stage, sequential deformation mechanism. This highlights the significant influence of the arrangement of tube lengths on the force–displacement characteristics and energy absorption performance of composite tubular energy-absorbing systems.

**Figure 7:**
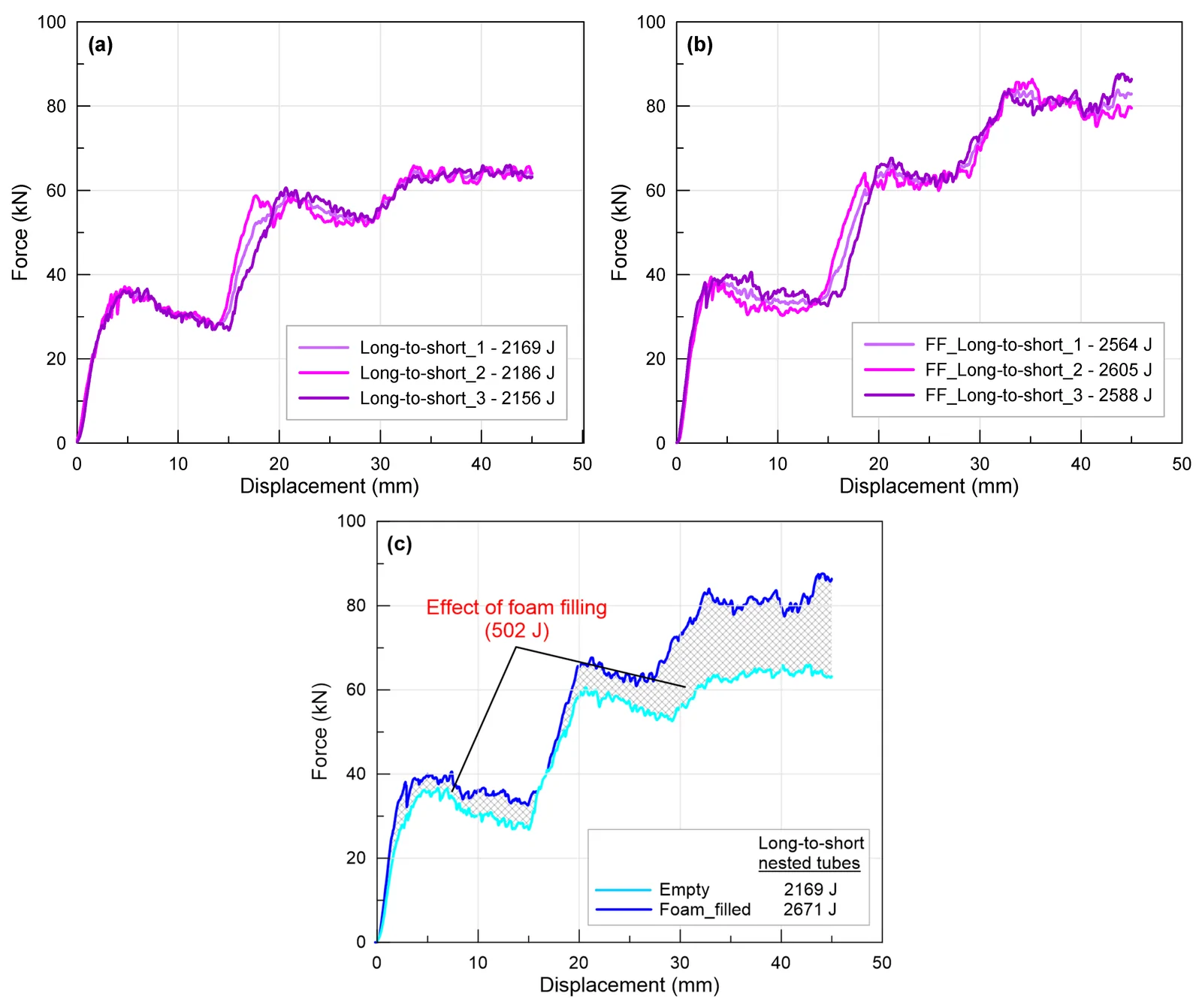
The force-displacement curves of the three repeated tests of (**a**) empty and (**b**) EPP foam-filled long-to-short nested GFRP tubular structures; (**c**) representative curves of empty and foam-filled nested samples show the foam filling effect in terms of absorbed energy (GFRP tubes were arranged concentrically, with the longer tube positioned on the outer side and the shorter tube placed on the inner side; tube 1: Ø77 × 100 mm, tube 2: Ø60 × 85 mm, tube 3: Ø37 × 70 mm).

Figure 7 illustrates the axial force-displacement responses of the long-to-short nested GFRP tube configuration subjected to quasi-static compressive loading: the three repeated tests of the empty (Figure 7a) and foam-filled (Figure 7b) specimens and representative curves of both (Figure 7c). The repeated curves are in good agreement, with absorbed energies of 2169, 2186, and 2156 J for the empty and 2564, 2605, and 2588 J for the foam-filled specimens. Similar to the short-to-long configuration and as described for Figure 5, the response is governed by a progressive, stepwise activation of the nested tubes, but in the reverse order. At the initial stage of deformation, the outer, longest tube (Ø77 × 100 mm) carries the load, resulting in a relatively high initial force compared to the short-to-long configuration. As compression proceeds, the subsequent engagement of the middle and inner, shorter tubes at approximately 15 and 30 mm results in further increases in the force level, producing multiple plateaus associated with successive crushing stages. This behavior indicates that, despite the reversed nesting order, the tubes are progressively crushed without abrupt drops in force. A clear distinction is observed between the empty and foam-filled configurations. The foam-filled system consistently exhibits higher force plateaus throughout the entire displacement range, which is attributed to the foam restraining the inward movement of the tube walls. Based on the group averages, the total absorbed energy increases from 2170 J for the empty configuration to 2586 J for the foam-filled configuration. This corresponds to an approximate 19% increase in energy absorption capacity due to foam filling. However, because of the added foam mass, the SEA remains practically unchanged (23.3 J/g and 23.0 J/g), and the maximum force increases from 65.6 kN to 87.0 kN. Compared to the short-to-long arrangement, the long-to-short arrangement absorbed 28% (empty) and 17% (foam-filled) more energy, whereas the equal-length arrangement absorbed the most energy overall. This trend is related to the early engagement of the largest-diameter outer tube, which is crushed over the full 45 mm stroke. When considered together with the previously analyzed short-to-long configuration, these results highlight the influence of the arrangement of tube lengths on energy absorption and force evolution.

**Figure 8:**
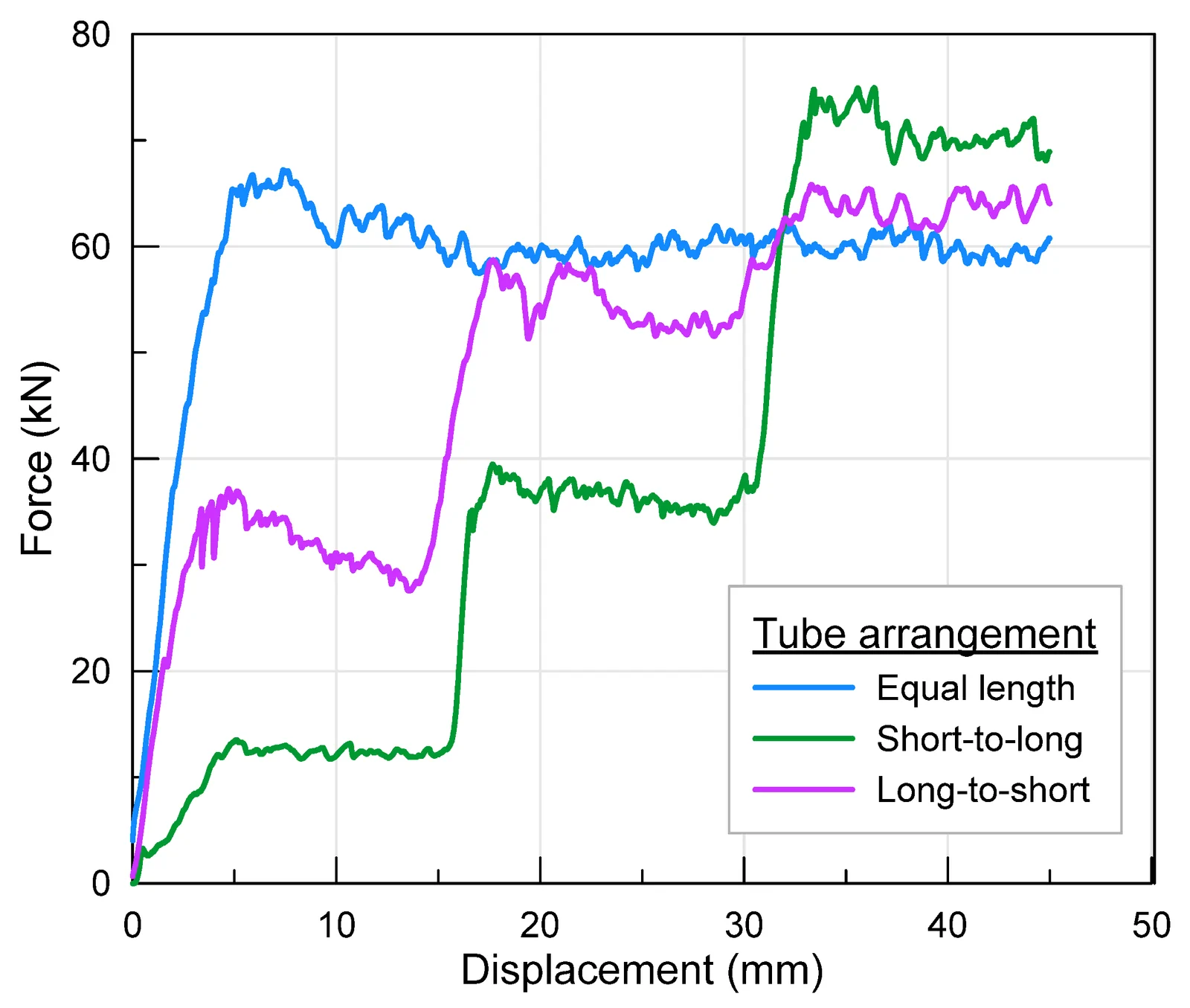
Comparison of representative force-displacement curves of the empty nested GFRP tubular structures with different arrangements.

Figure 8 compares representative force-displacement responses of the empty equal-length, short-to-long, and long-to-short nested GFRP tube configurations under quasi-static compression. The results show that tube arrangement strongly influences load evolution. The equal-length configuration exhibits an early rise to a nearly constant force level, indicating simultaneous engagement of all tubes and a stable, single-stage crushing response. In contrast, the short-to-long configuration shows a clear stepwise force evolution, with a low initial force followed by successive increases in load as the shorter tubes are activated. This arrangement provides the most gradual load escalation, distributing energy absorption over a wider displacement range. The long-to-short configuration exhibits a higher initial force due to the early engagement of the longest, largest-diameter tube, followed by further increases as inner tubes become active, resulting in higher force levels at larger displacements. Overall, configurations promoting sequential tube activation allow the force–displacement profile to be tailored, whereas the equal-length configuration provides the highest and most uniform force level. The short-to-long arrangement offers the most gradual force progression, while the long-to-short configuration achieves higher force levels and greater energy absorption than the short-to-long arrangement, highlighting nesting order as a key parameter in the design of nested GFRP tube systems.

Overall, across all three configurations, the equal-length tubes provide the most uniform and sustained crushing response with the highest energy absorption; the short-to-long configuration offers a highly progressive and controlled multi-stage deformation with reduced initial force levels; and the long-to-short configuration represents intermediate behavior. The long-to-short arrangement combines an initially high load-bearing capacity with staged deformation, resulting in moderate energy absorption and force levels that lie between those of the other two configurations. These observations indicate that the sequence of tube engagement plays a critical role in governing the force-displacement characteristics and crashworthiness performance of nested multi-tube GFRP structures. It should be noted that, within the same 45 mm stroke, the tubes of the staged configurations are crushed over different lengths; therefore, the differences in absorbed energy reflect both the nesting order and the amount of material engaged during the stroke.

**Figure 9:**
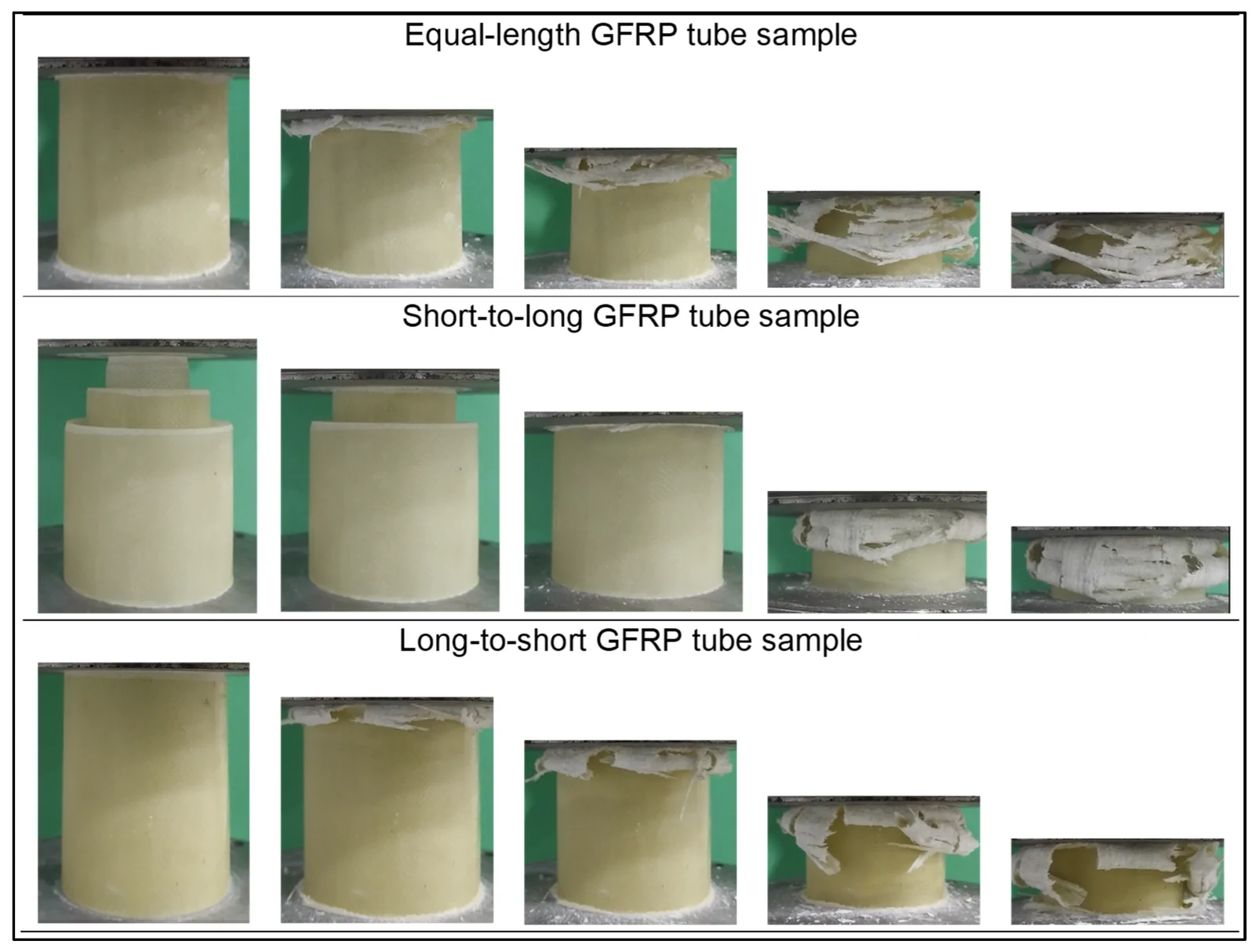
Crushing sequence of the empty nested GFRP tubes (equal-length, short-to-long and long-to-short configurations).

Figure 9 illustrates the crushing sequence of the empty nested tube configurations under axial compression. Despite differences in tube arrangement, all specimens failed by progressive end crushing initiated at the chamfered edges, a characteristic of composite tubes [51,52]. At the initial loading stage, all specimens remain largely undeformed while the load increases almost linearly. When the peak force is reached, damage initiates at the chamfered end in contact with the loading plate. After the initial peak, the tube walls are split into fronds that bend and splay inward and outward. Axial splits develop between the fronds, accompanied by matrix cracking (visible as stress whitening), delamination, and fiber fracture, and the crushed debris accumulates at the loaded end. In the equal-length configuration, the nested tubes engage simultaneously during compression, resulting in a relatively uniform deformation pattern and a single force plateau. For the short-to-long configuration, deformation initiates in the innermost, longest tube, and the middle and outer tubes are engaged after approximately 15 and 30 mm of displacement, respectively. The tubes are progressively crushed without global instability, and the force increases in discrete steps as the tubes are engaged one after another, consistent with the discussion in Figure 6; the low crush force efficiency of this arrangement reflects this stepwise force profile rather than an unstable crushing mode. In contrast, in the long-to-short configuration, the longer outer tube initially resists the compressive load, while the shorter inner tubes progressively participate in the deformation at later stages. This staged engagement produces a stepwise evolution of force without abrupt load drops. Within the 45 mm stroke, no densification was observed in the empty specimens. The energy absorption capability of the nested tube structures is therefore governed by the progressive crushing of the composite walls, the sequence of tube engagement, and the geometric arrangement.

Table 2 presents the axial crushing performance of nested tube configurations for the three repeated tests of each group, where E denotes empty nested tubes and FF represents EPP foam-filled nested tubes. In the equal-length configuration, the introduction of EPP foam results in a substantial improvement in energy absorption. The mean crushing force increases from 58.5 kN (E) to 78.6 kN (FF), corresponding to approximately a 34% increase. Similarly, the absorbed energy rises from 2630 J to 3537 J, representing a 34.5% improvement. The specific absorbed energy (SEA) increases from 27.5 J/g to 30.8 J/g, yielding a 12% enhancement, despite the increase in structural mass due to foam filling. The CFE shows a slight reduction from 0.86 to 0.82 (5% decrease), primarily due to the higher maximum force; however, the CFE remains high, indicating a uniform crushing response. In the short-to-long configuration, empty nested tubes exhibit the lowest energy absorption. The absorbed energy of the E specimens is limited to 1699 J, with a low CFE of 0.50, reflecting the stepwise force–displacement response. Foam filling increases the crushing resistance: F_mean_ increases from 37.7 kN to 49.1 kN, a ~30% increase, while the absorbed energy rises from 1699 J to 2208 J, a ~30% increase. Nevertheless, the SEA increases only from 19.8 J/g to 21.0 J/g (6%), and the CFE increases only marginally, from 0.50 to 0.52, since the force profile of this arrangement is governed by the staged engagement of the tubes. The long-to-short configuration exhibits a more uniform force response than the short-to-long configuration. For empty tubes, the absorbed energy reaches 2170 J, with a relatively high CFE of 0.74. When foam filling is introduced, F_mean_ increases from 48.2 kN to 57.5 kN, corresponding to a 19% increase, while the absorbed energy rises from 2170 J to 2586 J, indicating a 19% improvement. The SEA remains practically unchanged (23.3 J/g and 23.0 J/g); this change lies within the scatter of the individual results and shows that the additional mass introduced by the foam offsets the gain in total absorbed energy. Moreover, the CFE decreases from 0.74 to 0.66 (an 11% reduction), reflecting the higher maximum forces in the foam-filled specimens. In all configurations, foam filling increased the maximum force by 24–40%. The absorbed energies of the individual specimens differ by less than 1.5% from the group averages, confirming the good repeatability of the tests. Therefore, foam filling consistently increases the total absorbed energy and the mean crushing force, whereas its effect on the SEA depends on the nesting arrangement, and it increases the force transmitted to the protected structure.

The present results were obtained under quasi-static loading. Under impact, the strain-rate sensitivity of the epoxy matrix and of the EPP foam, inertia effects and changes in the failure mechanism may alter both the crushing forces and the SEA of composite tubes; the magnitude and even the sign of these changes depend on the material system and the failure mode [53,54]. Moreover, the higher maximum forces of the foam-filled specimens (+24–40%) would increase the decelerations transmitted to the protected structure. The suitability of the investigated configurations for automotive crash components, therefore, needs to be confirmed by dynamic tests and validated numerical models, which are planned as future work.

## 4. Conclusions

This study experimentally investigated the crashworthiness performance of nested GFRP tubes with and without EPP foam filling under quasi-static compression and the following results can be drawn:

The incorporation of EPP foam into nested GFRP tubes increased the mean crushing force and the absorbed energy in all configurations by approximately 34%, 30%, and 19% for the equal-length, short-to-long, and long-to-short configurations, respectively. In the equal-length configuration, foam filling increased the mean crushing force from 58.5 kN to 78.6 kN, improved the absorbed energy from 2630 J to 3537 J, and increased the specific energy absorption by about 12% despite the additional mass. However, the SEA increased by only 6% in the short-to-long configuration and remained practically unchanged in the long-to-short configuration, and the maximum force increased by 24–40% in all configurations.

The tube nesting arrangement was found to strongly influence the crushing response. In the staged configurations, the tubes were engaged at displacements equal to their height differences (15 and 30 mm), producing a stepwise force response (i). The equal-length configuration exhibited the highest overall energy absorption and a stable plateau response, while the short-to-long arrangement produced a sequential, multi-stage crushing behavior with the lowest initial force and a gradual load development. The long-to-short configuration, in which the largest-diameter tube was engaged first, absorbed more energy than the short-to-long configuration (iii) and showed intermediate behavior. Since the tubes of the staged configurations are crushed over different lengths within the same stroke, these differences reflect both the nesting order and the amount of material engaged.

Foam filling increased the force level at each crushing stage, consistent with the foam restraining the inward movement of the crushed wall material (ii). The gain in absorbed energy from foam filling should, however, be weighed against the added mass and the increased transmitted force. These quasi-static findings highlight the potential of foam-filled nested GFRP structures for lightweight, energy-absorbing applications, a potential that should be confirmed under dynamic loading.

Future work will focus on investigating the influence of different foam densities, tube geometries, and fiber stacking sequences and on evaluating the dynamic impact behavior and numerical modeling of nested composite tube systems to further optimize their crashworthiness.

## Figures and Tables

**Table 1:** Geometry and mass of the nested tube specimens (outer diameter × length of each tube in mm; masses are averages of three specimens; E: empty, FF: EPP foam-filled).

Configuration	Tube 1 (Outer)	Tube 2 (Middle)	Tube 3 (Inner)	Engagement of Tubes (mm)	Assembly Height (mm)	Mass E/FF (g)
Equal-length	Ø77 × 85	Ø60 × 85	Ø37 × 85	T1, T2, T3: 0	85	95.7/114.7
Short-to-long	Ø77 × 70	Ø60 × 85	Ø37 × 100	T3: 0; T2: 15; T1: 30	100	85.7/104.9
Long-to-short	Ø77 × 100	Ø60 × 85	Ø37 × 70	T1: 0; T2: 15; T3: 30	100	93.3/112.6

**Table 2:** Crashworthiness characteristics of the empty and EPP foam-filled nested GFRP tubular structures (three repeated tests per configuration).

Sample Configurations			F_mean_	F_max_	CFE (–)	Mass (g)	Absorbed Energy (J)	Specific Absorbed Energy (J/g)
Equal length	E*	1	58.2	67.2	0.87	95.8	2621	27.4
		2	58.7	69.2	0.85	95.7	2640	27.6
		3	58.4	68.1	0.86	95.7	2630	27.5
	Average		58.5	68.2	0.86	95.7	2630	27.5
	FF*	1	77.9	95.2	0.82	114.3	3507	30.7
		2	79.3	96.4	0.82	115.1	3569	31.0
		3	78.5	95.0	0.83	114.7	3534	30.8
	Average		78.6	95.5	0.82	114.7	3537	30.8
Short to long	E	1	37.5	74.8	0.50	85.3	1688	19.8
		2	38.0	77.8	0.49	86.0	1709	19.9
		3	37.8	76.0	0.50	85.7	1699	19.8
	Average		37.7	76.2	0.50	85.7	1699	19.8
	FF	1	48.3	93.4	0.52	104.4	2175	20.8
		2	49.8	95.5	0.52	105.5	2240	21.2
		3	49.1	95.0	0.52	104.9	2208	21.0
	Average		49.1	94.6	0.52	104.9	2208	21.0
Long to short	E	1	48.2	65.9	0.73	94.2	2169	23.0
		2	48.6	65.8	0.74	92.4	2186	23.7
		3	47.9	65.1	0.74	93.3	2156	23.1
	Average		48.2	65.6	0.74	93.3	2170	23.3
	FF	1	57.0	87.6	0.65	113.6	2564	22.6
		2	57.9	86.4	0.67	112.2	2605	23.2
		3	57.5	86.9	0.66	112.1	2588	23.1
	Average		57.5	87.0	0.66	112.6	2586	23.0

E*: indicates the empty tube samples, FF*: indicates the EPP foam-filled tube samples.

## Data Availability

The original contributions presented in this study are included in the article. Further inquiries can be directed to the corresponding author.
